# Clinical efficacy and tolerability of Esketamine: a case series

**DOI:** 10.1192/j.eurpsy.2022.2294

**Published:** 2022-09-01

**Authors:** C. Passani, N. Ragone, C.M. Congedo, B. Barbini, R. Zanardi, M.C. Cavallini, C. Colombo

**Affiliations:** 1 Università Vita Salute San Raffaele Hospital, Psichiatry, Mood Disorders Unit San Raffaele Turro, Milan, Italy; 2 IRCCS San Raffaele Scientific Institute, Psychiatry - Mood Disorders, Milano, Italy; 3 Università Vita-Salute San Raffaele, Psychiatry, Milano, Italy

**Keywords:** Depression, esketamine, treatment resistant depression

## Abstract

**Introduction:**

Esketamine is a novel antidepressant approved by the FDA in 2019 in the form of an intranasal spray, recommended for Treatment-Resistant Depression (TRD). The intranasal spray system appears to be more manageable than intravenous ketamine infusion. It contains ketamine’s S- isomer which is four-fold more potent for the NMDA receptor.

**Objectives:**

The aim of this case series is to describe our clinical experience in the use of Esketamine.

**Methods:**

6 TRD patients (3 men; 3 women) were recruited in San Raffaele Turro Hospital from March 2021. All patients (2 bipolar and 4 unipolar) were diagnosed with a Major Depressive Episode according to DSM-5 criteria, resistant to at least two antidepressants. Initially, Esketamine was administrated twice weekly for one month; afterward, it was administrated once weekly for a month; finally, it was administrated once weekly or every two weeks for a month. Clinical scales (HAM-D, YMRS, SSI, HAM-A, MADRS, CADSS) were administrated to assess symptoms and sides effects before and after each administration on a weekly basis.

**Results:**

Three patients out of six showed an improvement in depressive symptoms: two patients had remission (final HAM-D score < 8); one patient had a clinical response (final HAM-D score < 50 % respect baseline value). Three patients withdrew the treatment: two for perceived inefficacy, after 16 and 19 administrations, one for personal reasons.

**Conclusions:**

The use of Esketamine in our TRD patients showed good effectiveness and tolerability but randomized controlled clinical trials are needed to confirm our findings.

**Disclosure:**

No significant relationships.

